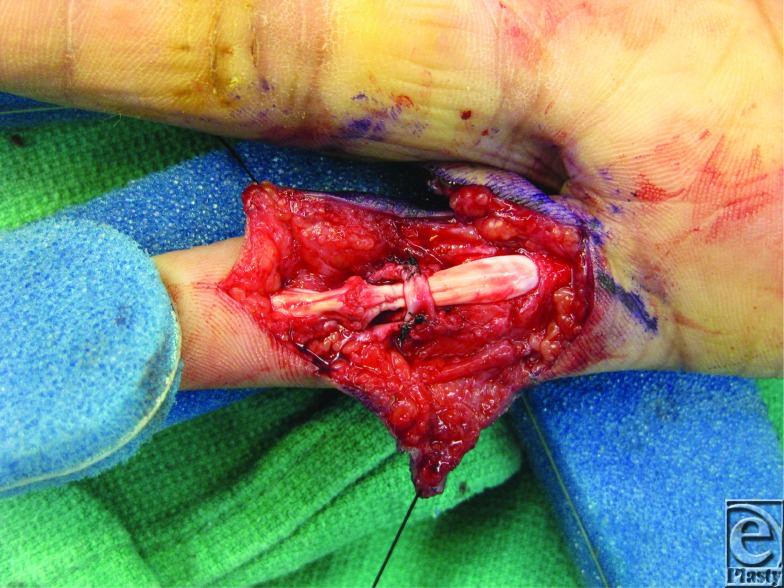# Zone II Flexor Tendon Injury

**Published:** 2013-01-25

**Authors:** Brenon Abernathie, Ramazi Datiashvili

**Affiliations:** Division of Plastic Surgery, Department of Surgery, New Jersey Medical School, University of Medicine and Dentistry of New Jersey, Newark

**Figure:**
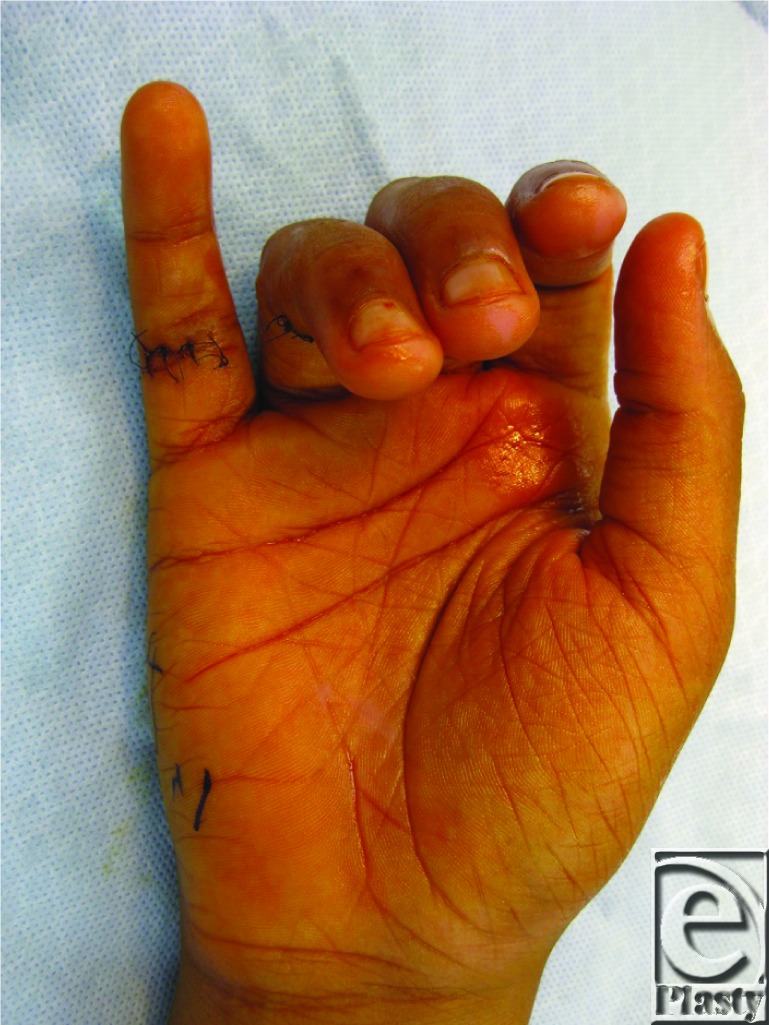


## DESCRIPTION

A 14-year-old adolescent boy presented 3 days after lacerating his hand with a knife. He complained of inability to flex his little finger and had decreased sensation on the ulnar aspect of this digit.

## QUESTIONS

1. **Describe the timing of flexor tendon repair.**
2. **What is the function of flexor tendon pulleys and which ones are essential?**
3. **When should isolated zone II flexor tendon injuries be mobilized after repair?**

## DISCUSSION

Bunnell, in 1918, coined the term “no man's land” to describe zone 2 in the hand because at that time it was felt that no man should attempt repair within this zone. While this belief is no longer a common practice, the ability to preserve the smooth gliding properties of both superficialis and profundus tendons within the narrow flexor sheath can be challenging for any hand surgeon. While tendon injuries may be repaired primarily at the time of initial presentation, tendon repair a few days to even 3 to 4 weeks later has been shown to yield outcomes close to those primary repairs done within a day of injury.

The pulley system of the digital flexor tendon consists of 5 annular (A1 through A5) and 3 cruciate (C1 through C3) pulleys responsible for maintaining anatomical course of tendons close to bones and phalangeal joints, thus optimizing the mechanical efficiency of digital flexion. The A2 and A4 pulley are the most efficacious in bowstringing prevention, and care should be taken to preserve or repair these structures if damaged. Sometimes, the A2 and A4 pulley may be an obstacle for the edematous tendon to glide through, possibly leading to rupture during postoperative motion exercise and thought should be given to partial release or reconstruction. However, reports and studies have indicated that incision of the entire A4 pulley and up to two thirds of the A2 pulley do not significantly affect tendon gliding when all other pulleys and sheath are intact.

After surgery, the hand is placed in a dorsal, extension blocking splint with the wrist at 20° to 30° flexion for approximately 2.5 weeks. Except in a few instances, such as noncompliant patients or concomitant fractures, postoperative mobilization should begin within 3 to 5 days to avoid restrictive adhesion formation. The Kleinert and Duran-Houser protocols are 2 common approaches to postoperative flexor tendon repair therapy consisting of active extension-passive flexion and only passive finger flexion methods, respectively.

In the case presented, our patient was found to have complete transection of the Flexor Digitorum Superficialis (FDS) and Flexor Digitorum Profundus (FDP) tendons as well as transection of the ulnar digital nerve. The injury to the FDS was found to be at the level of its insertion making the bone repair unfeasible, so repair of FDS was not attempted. The distal end of the FDP was unable to be passed under the A4 pulley, due to edema of the contused tendon, and the pulley was subsequently transected completely. The FDP was repaired with a modified Kessler suture and subsequent epitendinous repair. Because of prominent bowstringing, we reconstructed the A4 pulley using a segment of the FDS tendon. The ulnar nerve was repaired primarily and then the wound was closed and patient placed in a dorsal, extension-blocking splint with plans to begin therapy using the modified Kleinert protocol within 5 days after operation despite the digital nerve repair, as the nerve was repaired with minimal tension and would likely not be affected with therapy initiation. Three weeks postoperatively, he was able to flex his little finger to within 1 cm of the distal palmar crease.

## Figures and Tables

**Figure:**